# An exploratory analysis of associations of genetic variation with the efficacy of tocilizumab in severe COVID-19 patients. A pharmacogenetic study based on next-generation sequencing

**DOI:** 10.3389/fphar.2024.1426826

**Published:** 2024-09-13

**Authors:** Alejandro Durán-Sotuela, Jorge Vázquez-García, Sara Relaño-Fernández, Vanesa Balboa-Barreiro, Juan Fernández-Tajes, Francisco J. Blanco, Ignacio Rego-Pérez

**Affiliations:** ^1^ Grupo de Investigación en Reumatología (GIR), Instituto de Investigación Biomédica de A Coruña (INIBIC), Complexo Hospitalario Universitario de A Coruña (CHUAC) Sergas, Universidade da Coruña (UDC), A Coruña, Spain; ^2^ Unidad de Apoyo a La Investigación, Grupo de Investigación en Enfermería y Cuidados en Salud, Grupo de Investigación en Reumatología y Salud (GIR-S), Instituto de Investigación Biomédica de A Coruña (INIBIC), Complexo Hospitalario Universitario de A Coruña (CHUAC) Sergas, Universidade da Coruña (UDC), A Coruña, Spain; ^3^ Universidade da Coruña (UDC), Centro de Investigación de Ciencias Avanzadas (CICA), Grupo de Investigación en Reumatología y Salud (GIR-S), Departamento de Fisioterapia, Medicina y Ciencias Biomédicas, Facultad de Fisioterapia, Campus de Oza, A Coruña, Spain

**Keywords:** pharmacogenetics, tocilizumab, COVID-19, next-generation sequencing, interleukin

## Abstract

**Background:**

In the context of the cytokine storm the takes place in severe COVID-19 patients, the *Interleukin 6* (*IL6*) pathway emerges as one of the key pathways involved in the pathogenesis of this hyperinflammatory state. The strategy of blocking the inflammatory storm by targeting the *IL6* is a promising therapy to mitigate mortality. The use of Tocilizumab was recommended by the World Health Organization (WHO) to treat severe COVID-19 patients. However, the efficacy of Tocilizumab is variable. We hypothesize that the genetic background could be behind the efficacy of Tocilizumab in terms of mortality.

**Methods:**

We performed a targeted-next generation sequencing of 287 genes, of which 264 belong to a community panel of ThermoFisher for the study of genetic causes of primary immunodeficiency disorders, and 23 additional genes mostly related to inflammation, not included in the original community panel. This panel was sequenced in an initial cohort of 425 COVID-19 patients, of which 232 were treated with Tocilizumab and standard therapy, and 193 with standard therapy only. Selected genetic variants were genotyped by single base extension in additional 245 patients (95 treated with Tocilizumab and 150 non-treated with Tocilizumab). Appropriate statistical analyses and internal validation, including logistic regression models, with the interaction between Tocilizumab and genetic variants, were applied to assess the impact of these genetic variants in the efficacy of Tocilizumab in terms of mortality.

**Results:**

Age (*p* < 0.001) and cardiovascular disease (*p* < 0.001) are risk factors for mortality in COVID-19 patients. The presence of GG and TT genotypes at *IL10Rβ* (rs2834167) and *IL1β* (rs1143633) genes significantly associates with a reduced risk of mortality in patients treated with Tocilizumab (OR = 0.111; 95%CI = 0.015–0.829; *p* = 0.010 and OR = 0.378; 95%CI = 0.154–0.924; *p* = 0.028 respectively). The presence of CC genotype at *IL1RN* (rs2234679) significantly associates with an increased risk of mortality, but only in patients not treated with Tocilizumab (OR = 3.200; 95%CI = 1.512–6.771; *p* = 0.002). Exhaustive internal validation using a bootstrap method (B = 500 replicates) validated the accuracy of the predictive models.

**Conclusion:**

We developed a series of predictive models based on three genotypes in genes with a strong implication in the etiopathogenesis of COVID-19 disease capable of predicting the risk of mortality in patients treated with Tocilizumab.

## Background

On 31 December 2019, China reported to the World Human Organization (WHO) a series of cases of a strange pneumonia in Wuhan, Hubei Province, China. These cases of pneumonia were caused by a novel coronavirus, initially called 2019-nCoV (Phelan et al., 2020). On 30 January 2020, the outbreak of this disease, called Coronavirus disease 2019 (COVID-19), was declared a global public health emergency, being declared a global pandemic on March 2020 (Singh and Haq, 2021). Since then and until the time of writing this manuscript, this novel coronavirus, later renamed as severe acute respiratory syndrome coronavirus 2 (SARS-CoV-2), claimed over seven million lives worldwide (Arman et al., 2023).

A high percentage (∼80%) of patients that experienced COVID-19 disease were asymptomatic (with or without mild pneumonia), whilst 15% developed moderate pneumonia with hypoxemia, and 5% of patients suffered an extreme pneumonia capable of giving rise to an important acute respiratory distress syndrome (ARDS), organ failure or septic shock (Huang et al., 2020). In line with the latter, accumulating evidence suggests that a subgroup of patients with severe COVID-19 might experience the development of systemic complications associated to hypercytokinemia, hyperinflammation and the development of a cytokine storm (Alomair et al., 2023).

Cytokine storm is triggered by an excessive release of pro-inflammatory cytokines subsequently leading to immune dysregulation. In fact, sudden uncontrolled release of chemokines and pro-inflammatory cytokines is the most suitable definition for cytokine storm (Al-Kuraishy et al., 2023). Within this scenario, high levels of different inflammatory biomarkers, such as D-dimer, C-reactive protein (CRP), *Lactate dehydrogenase* (*LDH*), *Interleukin 1β* (*IL1β*) or *Interleukin 6* (*IL6*) have been proposed to be associated with the development of cytokine storm and, therefore, with COVID-19 severity, fatality and poor clinical outcomes (Vaninov, 2020; Farid et al., 2021). Considering that high levels of *IL6* had also been detected in other highly coronavirus-mediated infectious diseases, such as the Middle East respiratory syndrome (MERS) in 2012 or severe acute respiratory syndrome (SARS) in 2002, the *IL6* pathway emerges as one of the key pathways involved in the pathogenesis of this hyperinflammatory state (Li et al., 2023). Moreover, a lot of evidence accumulated suggesting that *IL6* is the most predictor cytokine in relation to COVID-19 severity and mortality (Ruan et al., 2020).

Taking into account the above mentioned, and considering the high risk of mortality in patients developing a severe COVID-19 progressing to ARDS, the strategy of blocking the inflammatory storm could serve as an efficacious treatment for severe COVID-19. Specifically, targeting of *IL6* could serve as a promising therapy to mitigate mortality (Li et al., 2023). Based on this, *IL6* receptor antagonists, such as Tocilizumab, emerged as one of the selected drugs to be applied on patients who were receiving systemic corticosteroid therapy and requiring oxygen therapy (mechanical ventilation or extracorporeal membrane oxygenation). Later, on 6 July 2021, the WHO strongly recommended the use of Tocilizumab in patients with severe COVID-19.

However, different meta-analysis of randomized clinical trials showed that the efficacy of Tocilizumab, as well as other *IL6* receptor antagonists, in different COVID-19 related features such as mortality, use of mechanical ventilation or length of intensive care unit (ICU) stay, is variable and unclear. Some of these studies found a benefit in mortality with Tocilizumab treatment (Godolphin et al., 2022; Piscoya et al., 2022; Yu et al., 2022; Ghosn et al., 2023), while others did not find significant differences on the risk of mortality between Tocilizumab and placebo groups (Boppana et al., 2022; Peng et al., 2022; Peng et al., 2023).

We speculate that genetic background, in addition to other potential risk factors, could be behind the efficacy of Tocilizumab. Therefore, this study aims to perform a pharmacogenetic approach in order to find out genetic variants potentially related to the efficacy of Tocilizumab, in terms of mortality, in patients with severe COVID-19 pneumonia. To carry out this study, we collected DNA samples from 670 severe COVID-19 patients in different hospitals in Spain, recruited between January 2020 and December 2021, which were subsequently subjected to an in-depth sequencing of a panel of 287 genes related to the immune response.

## Methods

**FIGURE 1:**
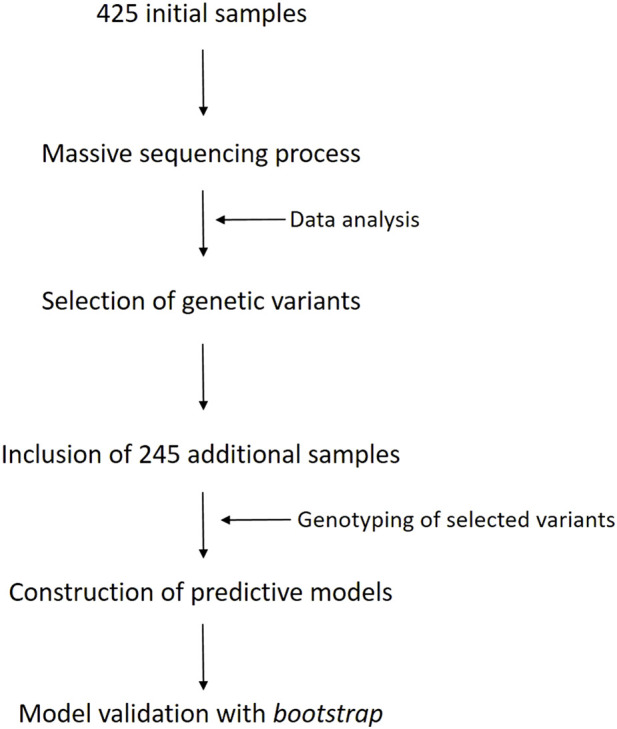
Workflow diagram of the different steps carried out in this work.

A scheme of the different steps of the methodology are shown in the workflow diagram described in Figure 1.

### Cohort description

For this study, we used data and samples of patients with Spanish origin from five Spanish hospitals, collected through the National network of biobanks of Spain: Hospital de Bellvitge (n = 28), Hospital Ramón y Cajal (n = 31), Hospital Puerta de Hierro (n = 36), Hospital Universitario de A Coruña (n = 562) and Hospital Clínico de Santiago (n = 13). In total, there were 670 patient samples, of which 327 were treated with Tocilizumab (intravenous infusion of 8 mg/kg body weight) and standard therapy (mostly corticosteroids and/or antivirals), and 343 were treated with standard therapy only. All these patients suffered from severe COVID-19 pneumonia, characterized by multifocal pneumonia, dyspnea, and oxygen saturation < 90%.

A series of clinical data from these patients were provided by the different hospitals, including: sex, age, occurrence of cardiovascular disease (including coronary artery disease, acute myocardial infarction, heart failure or atrial fibrillation), diabetes, arterial hypertension status, oxygen support and treatment. The outcome of this study was the risk of mortality due to COVID-19-derived complications.

All the clinical centers involved in the recruitment of these patients have made provisions to ensure the safety, confidentiality and ethical treatment of study participants according to the Declaration of Helsinki. In accordance with the ethical requirements, all the participants signed an informed consent. This study was approved by the Galician research ethics committee (registration code 2020/215).

### Targeted-next generation sequencing

For this study, we used the Ampliseq DNA Panel IAD207654_182 (ThermoFisher Scientific), a NGS-based panel where the individuals exact genotype at all sites is read out across exonic regions of interest. The panel consisted of a mixture of 264 genes belonging to a community panel of ThermoFisher for the study of genetic causes of primary immunodeficiency disorders, based on extensive curation of peer-reviewed literature and disease research databases, and 23 additional genes mostly related to inflammation and not included in the original community panel (Supplementary Table 1). For the sequencing of these 287 genes, the panel consisted of two primer pools (2,842 primer pairs each), 572,241 kb in size and comprised 5,241 amplicons. The in-depth sequencing of this gene panel was performed in an initial subset of 425 genomic DNA (gDNA) samples, of which 232 were samples from patients treated with Tocilizumab and standard therapy, and 193 corresponded to samples from patients treated with standard therapy only.

The preparation of libraries and the subsequent in-depth sequencing of the samples was carried out in the Ion S5XL/Ion Chef platform (ThermoFisher Scientific). A total of 100 ng of DNA in a final volume of 15 µL were used to construct the barcoded DNA libraries using the Precision ID DL8 Kit (ThermoFisher Scientific) following manufacturer´s recommendations. Up to eight libraries per run were automatically prepared by the Ion Chef, resulting in a final pool of 700 μL at 100p.m. in one tube. Since the maximum number of barcodes available using this kit is 32, we performed four independent runs on the Ion Chef to generate four pools of libraries (8 per pool). Finally, each set of (8 × 4) 32 libraries was pooled into a single tube in a final volume of 25 µL and normalized to 70p.m., ready for templating and sequencing on the Ion S5XL platform.

Generated libraries were ready for template preparation through clonal amplification on the Ion Chef system following manufacturer´s recommendations. This process concludes with the automatic loading of the samples into the Ion 550 sequencing chip. As a result, approximately 150 million reads per chip were obtained, with an average read depth of 700X.

### Analysis of sequencing data

The obtained reads were cleaned and aligned to GrCh37 Human reference sequence using the Torrent Suite 5.18.1 (ThermoFisher Scientific). Variant calling was performed through an adapted pipeline for Ampliseq libraries using a low stringency mode in Torrent Variant Caller plug-in (TVC) (Generic - S5/S5XL (550) - Germ Line - Low Stringency). Each variant was examined based on: read depth (≥ 100), Phil´s Read Editor (PHRED) score (≥ 100) and variant frequency (≥ 0.20). The different genomic variant call formats (vcfs) were aggregated using *bcftools* v1.16 to obtain a multisample vcf containing all the samples in the study. This multisample vcf was filtered to rule out monoallelic variants and variants with a minor allele frequency (MAF) lower than 0.01. Potential pathogenicity of detected variants was assessed using PolyPhen (Polymorphism Phenotyping), Sift (Sorting Intolerant From Tolerant), Revel (Rare Exome Variant Ensemble Learner) and Caad (Combined Annotation Dependent Depletion), in addition to the online tool ClinVar (https://www.ncbi.nlm.nih.gov/clinvar/).

### Statistical analyses of DNA sequencing data

All the statistical analyses were performed using R software v3.6.3 (The R Foundation for Statistical Computing) and IBM-SPSS software, release 29 (IBM, Armonk, NY, United States).

The initial multisample vcf of 425 patients was converted to plink format using *plink* v1.9 and then performed a logistic regression using risk of mortality as outcome. We included an interaction in the model between the allelic dosage additive effect and Tocilizumab (ADDxTOCI term). The results for the ADDxTOCI term for each variant was exported and converted to a bed format using custom scripts and further annotated using *SnpEff* v5.2.

For the analysis of the complete cohort of 670 patients, a binary logistic regression model was constructed using, as dependent variable, the risk of mortality and, as confounding factors, sex, age, cardiovascular disease and the interaction between Tocilizumab and each of the three selected genotypes. Sensitivity, specificity and negative and positive predictive values were provided as measures of model performance. The optimal cut-off value was chosen using the Youden index. As measures of the performance of the proposed models, the percentage of success and the area under the curve (AUC) are provided. The obtained AUCs were compared using the DeLong´s test for two correlated receiver operating characteristic (ROC) curves. Additionally, the Brier score was also calculated as a measure of the precision of the probabilistic predictions. This score evaluates whether the model adequately predicts whether the probability predicted by the model is actually correct.

In addition, as a necessary part of model development, we have relied on the TRIPOD (Transparent reporting of a multivariate prediction model for individual prognosis or diagnosis) statement to perform an exhaustive internal validation, using the bootstrap validation method (B = 500 replicates). Thus, the above-obtained model was evaluated by computing the overall accuracy for match outcome via bootstrap corrected AUC. For this purpose, both *pROC* and *boot* packages from R software were used.

As an additional measure, the population-attributable fraction (PAF) of death was also calculated. This parameter is the proportional reduction in mortality that would occur if exposure to a specific risk factor was reduced to an alternative ideal exposure scenario. In this case, this score represents the proportion of deceased patients in the population that is due to the administration of Tocilizumab, alone or in combination with different genotypes. For this purpose, *twoxtwo* package from R software was used.

### Single base extension (SBE) assay

To confirm the presence of the selected variants, as well as to complete the genotyping of these variants in the remaining 245 samples (95 Tocilizumab and 150 non-Tocilizumab), we used the SBE assay on a SeqStudio genetic analyzer system (ThermoFisher Scientific). Briefly, an initial polymerase chain reaction (PCR) was performed to amplify the DNA fragments containing the informative single nucleotide polymorphism (SNP). The resulting PCR fragments were purified with ExoSap-It (ThermoFisher Scientific) and subsequently subjected to the SBE reaction following manufacturer´s recommendations. Purified SBE products using Fast-AP (ThermoFisher Scientific) were loaded into a SeqStudio genetic analyzer to visualize the SNPs. Primers used for this assay are available upon request.

This technique was also applied to confirm the selected variants in a 20% of randomly selected samples from the initial subset of 425 patients.

## Results

### Descriptive analysis of the study population

The initial univariate descriptive analysis of the 670 patients revealed some significant differences in terms of mortality (Table 1). Among the different collected covariables, only age above 69 years (81.7% vs. 39.4%; *p* < 0.001), higher frequency of cardiovascular disease (25.3% vs. 9.6%; *p* < 0.001) and arterial hypertension (60% vs. 46.6%; *p* = 0.003) appeared significantly associated as risk factors for mortality due to COVID-19 derived complications.

**TABLE 1:** Clinical characteristics of the study population stratified by mortality.

Clinical characteristic	Deceased (N = 175)	Non-deceased (N = 495)	*p*-value	Total (N = 670)
Median age at baseline			<0.001*	
Above 69 years	143 (81.7)	195 (39.4)		338 (50.4)
Below 69 years	32 (18.3)	300 (60.6)		332 (49.6)
Gender			0.711^#^	
Male	112 (64.0)	309 (62.4)		421 (62.8)
Female	63 (36.0)	186 (37.6)		249 (37.2)
Cardiovascular disease			**<0.001** ^**#**^	
Yes	43 (25.3)	42 (9.6)		85 (14.0)
No	127 (74.7)	394 (90.4)		521 (86.0)
Arterial hypertension			**0.003** ^**#**^	
Yes	102 (60.0)	203 (46.6)		305 (50.3)
No	68 (40.0)	233 (53.4)		301 (49.7)
Type-2 diabetes			0.668^#^	
Yes	37 (21.8)	102 (23.4)		139 (22.9)
No	133 (78.2)	334 (76.6)		467 (77.1)
Oxygen support			0.541^#^	
Yes	39 (37.5)	106 (34.2)		145 (35.0)
No	65 (62.5)	65 (62.5)		269 (65.0)
Tocilizumab			0.438^#^	
Yes	81 (46.3)	246 (49.7)		327 (51.2)
No	94 (53.7)	249 (50.3)		343 (51.2)

Values are mean ± standard deviation or number of patients with percentage in parentheses; (*) Mann-Whitney U test for comparison between deceased and non-deceased patients; (#) chi-square test; significant *p*-values are in bold

### Identification of differential DNA variants

A total of 8,933 variants (including those that were not significant) were identified in the initially sequenced 425 samples,. However, the statistical analyses showed a set of potential polymorphisms whose interaction with Tocilizumab was nominally statistically significant in terms of decreased mortality (Supplementary Table 2). Of all of them, we selected three polymorphisms: rs2834167 (*p* = 0.012), a missense variant (Aaa/Gaa) at *Interleukin 10 receptor β* (*IL10Rβ*) gene; rs1143633 (*p* = 0.008), an intron variant (C/T) at *IL1β*; and the tag SNP rs2234679 (*p* = 0.008), a 5′ untranslated region (UTR) variant (G/C) at *Interleukin one receptor antagonist* (*IL1RN*) gene.

### Analysis of DNA variants in the complete cohort and predictive models

The univariate analysis of genotype frequencies for the three selected polymorphisms in the complete cohort of 670 patients (Table 2) showed a significant association of genotypes GG (vs. non-GG) and TT (vs. non-TT) of rs2834167 and rs1143633 respectively, with a lower risk of mortality in patients treated with Tocilizumab (OR = 0.111; 95%CI = 0.015–0.829; *p* = 0.010 and OR = 0.378; 95%CI = 0.154–0.924; *p* = 0.028 respectively). On the other hand, the risk of mortality was higher in those COVID-19 patients harboring the CC genotype of rs2234679 that were not treated with Tocilizumab (OR = 3.200; 95%CI = 1.512–6.771; *p* = 0.002); however, this latter difference was not present in the group of patients treated with Tocilizumab.

**TABLE 2:** Frequency distribution of genotypes between deceased and non-deceased severe COVID-19 patients stratified by Tocilizumab uptake.

Tocilizumab							
SNP	Genotype	Deceased (N = 81)	Non-deceased (N = 246)	Total (N = 327)	*p*-value	OR	95% CI
rs2834167	GG	1 (1.2)	25 (10.2)	26 (8.0)	**0.010**	0.111	0.015–0.829
	non-GG	80 (98.8)	221 (89.8)	301 (92.0)			
rs1143633	TT	6 (7.4)	43 (17.5)	49 (15.0)	**0.028**	0.378	0.154–0.924
	non-TT	75 (82.6)	203 (82.5)	278 (85.0)			
rs2234679	CC	7 (8.6)	17 (6.9)	24 (7.3)	0.604	1.274	0.509–3.192
	non-CC	74 (91.4)	229 (93.1)	303 (92.7)			

Non-Tocilizumab							
SNP	Genotype	Deceased (N = 94)	Non-deceased (N = 249)	Total (N = 343	*p*-value	OR	95% CI
rs2834167	GG	8 (8.5)	20 (8.0)	28 (8.2)	0.885	1.065	0.452–2.508
	non-GG	86 (91.5)	229 (92.0)	315 (91.8)			
rs1143633	TT	18 (19.1)	33 (13.3)	51 (14.9)	0.171	1.550	0.825–2.914
	non-TT	76 (80.9)	216 (86.7)	292 (85.1)			
rs2234679	CC	16 (17.0)	15 (6.0)	31 (9.0)	**0.002**	3.200	1.512–6.771
	non-CC	78 (83.0)	234 (94.0)	312 (91.0)			

Values are number of patients with percentage in parentheses; OR: odds ratio; CI: confidence interval; significance *p*-values are in bold

Next, we performed a series of regression logistic models including both clinical and genetic variables, as well as the interaction between Tocilizumab and each of the genotypes (Table 3). Because of the strong collinearity among diabetes, arterial hypertension and cardiovascular disease, of these three, only cardiovascular disease was considered in the predictive model, in addition to sex and age. Besides, oxygen support was also removed from the models due to the lack of information for a considerable proportion of patients.

**TABLE 3:** Multivariable logistic regression models to predict the effect of the three genotypes on the efficacy of Tocilizumab in severe COVID-19 patients and results of the bootstrap validation strategy.

Developed model for rs2834167 genotypes (*IL10Rβ* gene)					
Variable	B	SE	OR	95% CI	*p*-value
Gender (male)	0.076	0.211	1.079	0.715–1.639	0.718
Age (median)	1.744	0.229	5.721	3.694–9.097	**<0.001**
Cardiovascular disease	0.840	0.262	2.318	1.389–3.884	**0.001**
Tocilizumab (Yes)	−2.439	1.159	0.087	0.004–0.619	**0.035**
rs2834167.GG vs. AA	0.327	0.532	1.387	0.467–3.846	0.539
rs2834167.GG vs. AG	−0.029	0.541	0.971	0.322–2.747	0.957
Tocilizumab*rs2834167.GG vs. AA	−2.595	1.191	0.074	0.003–0.573	**0.029**
Tocilizumab*rs2834167.GG vs. AG	−2.304	1.200	0.099	0.005–0.784	0.055
Sensitivity	Specificity	NPV	PPV	% success	Brier score
0.847 (0.788–0.900)	0.578 (0.530–0.624)	0.906 (0.874–0.936)	0.439 (0.407–0.472)	65.3%	0.169
Bootstrap validation strategy for rs2834167 genotypes					
Model		AUC		95% CI	
Developed model		0.751		0.709–0.792	
Bootstrap corrected (B = 500)		0.732		0.694–0.771	

Developed model for rs1143633 genotypes (IL1β gene)					
Variable	B	SE	OR	95% CI	*p*-value
Gender (male)	0.108	0.210	1.114	0.739–1.689	0.608
Age (median)	1.802	0.231	6.061	3.904–9.664	**<0.001**
Cardiovascular disease	0.771	0.259	2.162	1.301–3.599	**0.003**
Tocilizumab (Yes)	−1.323	0.572	0.266	0.081–0.789	**0.021**
rs1143633.TT vs. CC	0.445	0.405	1.560	0.697–3.436	0.272
rs1143633.TT vs. CT	0.067	0.409	1.069	0.473–2.370	0.870
Tocilizumab*rs1143633.TT vs. CC	−1.792	0.652	0.167	0.045–0.580	**0.006**
Tocilizumab*rs1143633.TT vs. CT	−1.102	0.644	0.332	0.089–1.140	0.087
Sensitivity	Specificity	NPV	PPV	% success	Brier score
0.847 (0.794–0.900)	0.578 (0.532–0.624)	0.907 (0.874–0.937)	0.439 (0.408–0.471)	65.3%	0.170
Bootstrap validation strategy for rs1143633 genotypes					
Model		AUC		95% CI	
Developed model		0.752		0.711–0.793	
Bootstrap corrected (B = 500)		0.735		0.696–0.777	

Developed model for rs2234679 genotypes (*IL1RN* gene)					
Variable	B	SE	OR	95% CI	*p*-value
Gender (male)	0.057	0.211	1.058	0.701–1.607	0.789
Age (median)	1.755	0.230	5.786	3.733–9.211	**<0.001**
Cardiovascular disease	0.797	0.258	2.218	1.337–3.686	**0.002**
Tocilizumab (Yes)	−0.979	0.660	0.376	0.099–1.339	0.138
rs2234679.CC vs. GG	1.113	0.480	3.039	1.202–8.000	**0.020**
rs2234679.CC vs. GC	1.416	0.492	4.115	1.592–11.111	**0.004**
Tocilizumab*rs2234679.CC vs. GG	−0.988	0.717	0.372	0.088–1.486	0.168
Tocilizumab*rs2234679.CC vs. GC	−1.028	0.736	0.358	0.082–1.484	0.163
Sensitivity	Specificity	NPV	PPV	% success	Brier score
0.847 (0.788–0.793)	0.578 (0.530–0.626)	0.907 (0.875–0.937)	0.440 (0.409–0.473)	65.3%	0.169
Bootstrap validation strategy for rs2234679 genotypes					
Model		AUC		95% CI	
Developed model		0.753		0.712–0.794	
Bootstrap corrected (B = 500)		0.735		0.695–0.774	

B: regression coefficient; SE: standard error; OR: odds ratio; CI: confidence interval; AUC: area under the curve; NPV: negative predictive value; PPV: positive predictive value; (*) significant *p*-values are in bold

The model including the interaction between the GG genotype of rs2834167 at *IL10Rβ* gene and Tocilizumab (Table 3) confirmed the associations of age above 69 years (OR = 5.721; 95%CI = 3.694–9.097; *p* < 0.001) and cardiovascular disease (OR = 2.318; 95%CI = 1.389–3.884; *p* = 0.001) with the increased risk of mortality. Interestingly, the interaction between GG genotype of rs2834167 and Tocilizumab associated with a significant reduction of mortality when compared with patients harboring the AA genotype (OR = 0.074; 95%CI = 0.003–0.573; *p* = 0.029), and bordered on the statistical significance when compared with patients with the AG genotype (OR = 0.099; 95%CI = 0.005–0.784; *p* = 0.055). The AUC for this predictive model was 0.751 (95%CI: 0.709–0.792). This model showed a sensitivity of 0.847 (0.788–0.900), a specificity of 0.578 (0.530–0.624) as well as a negative and positive predictive values of 0.906 (0.874–0.936) and 0.439 (0.407–0.472) respectively.

The next model included the interaction between the TT genotype of rs1143633 at *IL1β* gene and Tocilizumab (Table 3). This model also confirmed the associations of age above 69 years (OR = 6.061; 95%CI = 3.904–9.664; *p* < 0.001) and cardiovascular disease (OR = 2.162; 95%CI = 1.301–3.599; *p* = 0.003) with the increased risk of mortality. In addition, the model also showed that the administration of Tocilizumab in patients harboring the TT genotype of rs1143633 associated with a decreased risk of mortality compared to patients harboring the CC genotype (OR = 0.167; 95%CI = 0.045–0.580; *p* = 0.006). The AUC of this model was 0.752 (95%CI: 0.711–0.793). This model showed a sensitivity of 0.847 (0.794–0.900), a specificity of 0.578 (0.532–0.624) as well as a negative and positive predictive values of 0.907 (0.874–0.937) and 0.439 (0.408–0.471) respectively.

The third model included the interaction between the CC genotype of rs2234679 at *IL1RN* gene and Tocilizumab (Table 3). The model maintained the association of age above 69 years (OR = 5.786; 95%CI = 3.733–9.211; *p* < 0.001) and cardiovascular disease (OR = 2.218; 95%CI = 1.337–3.686; *p* = 0.002) with increased mortality. Interestingly, this model also showed that the presence of CC genotype significantly associated with an increased risk of mortality, when compared both with patients with GG (OR = 3.039; 95%CI = 1.202–8.000; *p* = 0.020) and GC (OR = 4.115; 95%CI = 1.592–11.111; *p* = 0.004) genotypes; however, this association was not observed in patients with CC genotype that were treated with Tocilizumab. The AUC of this model was 0.753 (95%CI: 0.712–0.794). This model showed a sensitivity of 0.847 (0.788–0.793), a specificity of 0.578 (0.530–0.626) as well as a negative and positive predictive values of 0.907 (0.875–0.937) and 0.440 (0.409–0.473) respectively.

**FIGURE 2:**
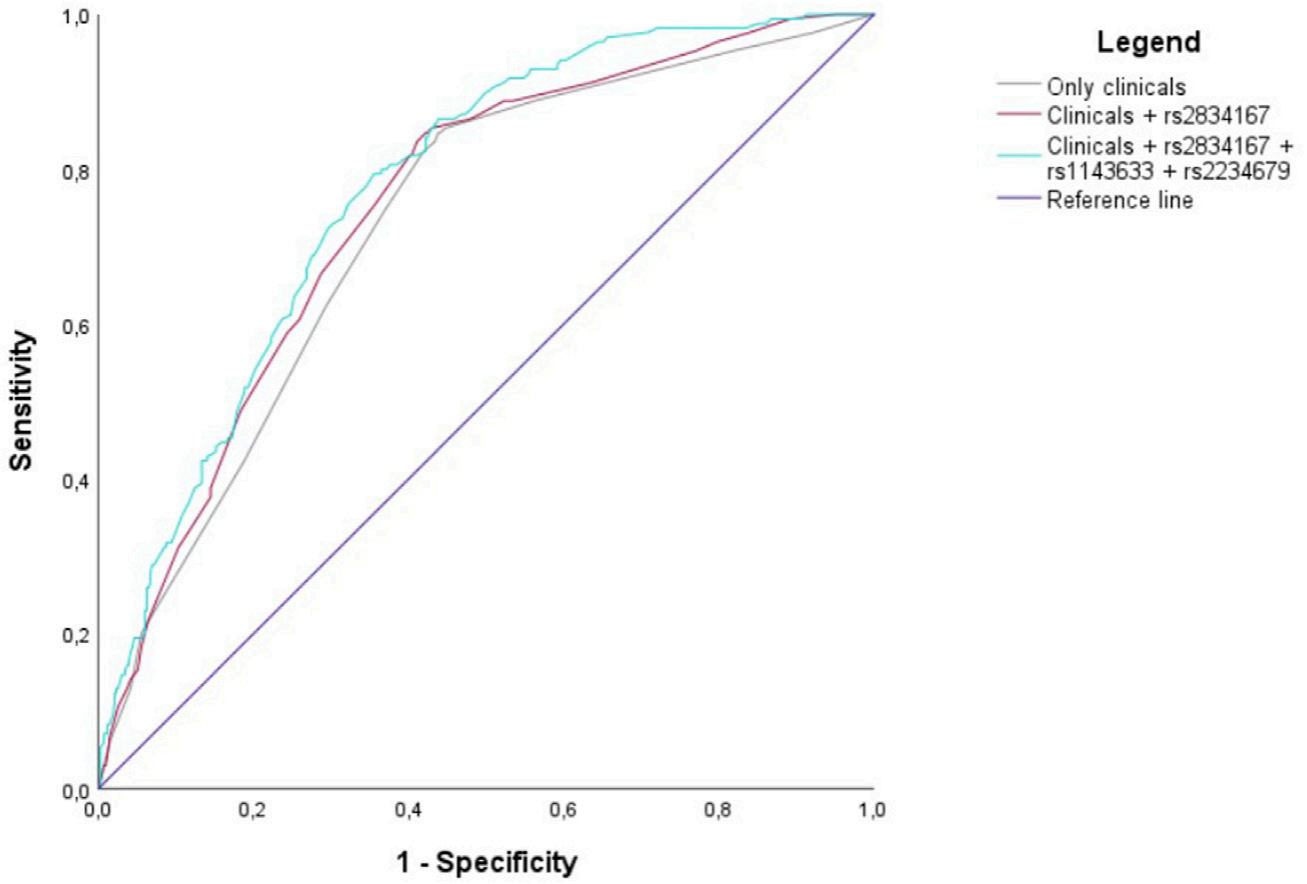
Receiver operating characteristic curves (AUCs) of three different scenarios: Clinical variables only (grey line), clinical variables and *IL10Rβ* genotype (brown line), and clinical variables and all the three genotypes (blue line).

For the three aforementioned models, the percentage of success was 65.3% and the Brier score was close to zero (between 0.169 and 0.170), indicating a good level of model calibration (Table 3). Interestingly, by including all three genotypes in a single regression model, the AUC reached a value of 0.775 (95%CI: 0.737–0.814), significantly improving the predictive capability when compared to both the model including only clinical variables (AUC = 0.730) (*p* = 0.001) and the model including clinical variables and *IL10Rβ* genotype (AUC = 751) (*p* = 0.030). The comparison between the clinical model (gender, age, cardiovascular disease and Tocilzumab) and the model with clinical variables and *IL10Rβ* genotype bordered on the statistical significance (*p* = 0.065) (Figure 2).

We additionally conducted a gene-gene interaction analysis among the three genotypes. However, we did not detect significant interactions that could suggest the existence of epistasis (data not shown, but available upon request).

To quantify the effect of Tocilizumab, alone or in combination with the identified genotypes, we calculated the PAF. Throughout this parameter, we can estimate the proportion of deceased patients due to different exposures. The results concluded that patients with the rs2834167 GG genotype treated with Tocilizumab show a significant 76.92% reduction in their mortality rate compared with rs2834167non-GG patients treated with Tocilizumab (1.37%). Similarly, patients with the rs1143633 TT genotype treated with Tocilizumab show a significant 48.98% reduction in their mortality rate compared with rs1143633 non-TT genotype (1.84%). The rest of exposures did not show statistical reductions in the mortality rate (Table 4).

**TABLE 4:** Population attributable fraction (PAF) at different exposures.

Exposures	Estimate	95%CI low	95%CI high
Tocilizumab only	5.16	0	18.20
Tocilizumab in patients with rs2834167 GG genotype	76.92*	35.5	100
Tocilizumab in patients with rs2834167 non-GG genotype	1.37	0	14.7
Tocilizumab in patients with rs1143633 TT genotype	48.98*	16.6	81.4
Tocilizumab in patients with rs1143633 non-TT genotype	1.84	0	12.2
Tocilizumab in patients with rs2234679 CC genotype	30.26	0	65.1
Tocilizumab in patients with rs2234679 non-CC genotype	1.18	0	15.2

PAF: population attributable fraction; CI: confidence interval; (*) *p*-value < 0.05

### Internal validation of the predictive models

These three regression models were internally validated using a bootstrap validation approach (B = 500 replicates) (Table 3). The accuracy of the models was measured through the AUC value. The corrected AUC for each of the three models, after the validation procedure, was 0.732 (95%CI: 0.694–0.771) for the model including the interaction between Tocilizumab and GG genotype of rs2834167 at *IL10Rβ* gene; 0.735 (95%CI: 0.696–0.777) for the model including the interaction between Tocilizumab and TT genotype of rs1143633 at *IL1β* gene; and 0.735 (95%CI: 0.695–0.774) for the model with the interaction between Tocilizumab and CC genotype of rs2234679 at *IL1RN* gene.

## Discussion

In this work, we carried out a pharmacogenetic study in 670 severe COVID-19 patients to check the efficacy of Tocilizumab in terms of reduced mortality. The results obtained show that the presence of a series of polymorphisms at *IL10Rβ* (rs2834167), *IL1β* (rs1143633) and *IL1RN* (rs2234679) genes significantly associates with a reduced risk of mortality in patients treated with Tocilizumab. Specifically, those patients carrying the GG genotype at *IL10Rβ*, or the TT genotype at *IL1β* gene, show an association with a lower risk of mortality when they are treated with Tocilizumab. On the other hand, those COVID-19 patients with the CC genotype at *IL1RN* associated with a significantly higher risk of mortality, but this latter association disappears when they are treated with Tocilizumab.

Among the three selected polymorphisms, only the SNP rs2834167 at *IL10Rβ* gene consists on a missense mutation (A/G) that leads to a Lysine to Glutamic acid change (K47E). This missense variant is reported in ClinVar (https://www.ncbi.nlm.nih.gov/clinvar/) as a benign variant protecting against inflammatory bowel disease, as well as a risk factor for susceptibility to Hepatitis B virus (Frodsham et al., 2006). In addition, some studies have reported different associations with both susceptibility and clinical manifestations of systemic lupus erythematosus (SLE) (Grk et al., 2023), as well as overweight (Maculewicz et al., 2022), hypertension in patients with ischemic stroke (Park et al., 2013) and poor prognosis of multiple myeloma patients treated with thalidomide and/or bortezomib (Kasamatsu et al., 2017). All these associations suggest that this polymorphism bears functional significance.

*IL10Rβ* is located in a class II cytokine receptor gene cluster, on chromosome 21q22, together with *Interferon-α receptor 1* (*IFNAR1*), *2* (*IFNAR2*) and *Interferon-γ receptor 2* (*IFNGR2*) (Hikami et al., 2008). This molecule acts as a receptor for members of the *IL10* family of cytokines that are essential in the modulation process of host defense mechanisms to limit, especially in epithelial cells, the damage caused by bacterial and viral infections (Ouyang et al., 2011). In terms of the involvement of this gene in the development of COVID-19 disease, a recent work consisted on a translational genomics approach, identified *IL10Rβ* as a top candidate gen target for COVID-19 susceptibility (Voloudakis et al., 2022). The authors conclude that overexpression of *IL10Rβ* gene in COVID-19 patient blood is associated with worse outcomes and increased viral load and activation of disease-relevant molecular pathways (Voloudakis et al., 2022). These findings would be in agreement with those reported by Shivram and collaborators in another study aimed to characterize the molecular effects of Tocilizumab treatment in COVID-19 patients by analyzing transcriptomic and proteomic data. In patients with severe COVID-19 who experienced a poor prognosis or even died, these authors reported an increased expression of inflammatory and antiviral pathways, in which the *IL10* family of cytokines is a key determinant (Shivram et al., 2023). All of the above mentioned could explain, at least in part, the association of this polymorphism with the increased efficacy of Tocilizumab in terms of decreased mortality, since the GG genotype has been reported to decrease the mRNA expression level of *IL10Rβ* (Frodsham et al., 2006).

In addition to other pro-inflammatory cytokines, such as *Tumor necrosis factor α* (*TNFα*) and *IL6*, *IL1β* is also related to COVID-19 pathogenesis (Del Valle et al., 2020). Specifically, monocytes from patients infected with SARS-CoV-2 present a *Nod-like receptor protein 3* (*NLRP3*) inflammasome activation and, therefore, spontaneous secretion of *IL1β* (Bertoni et al., 2022). Inflammasome is a multiprotein complex that regulates the activation of *Caspase-1* and the production and secretion of *IL1β* as well as other potent pro-inflammatory cytokines. *IL1β* secretion contributes to the expression of other cytokines, such as *TNFα* and *IL6*, leading to a hypercytokinemia state and, therefore, playing a capital role in inflammatory syndromes characterized by the occurrence of cytokine storm, such as severe COVID-19 pneumonia (Jamilloux et al., 2020; Napodano et al., 2023). Interestingly, Tocilizumab reduces not only inflammasome activation, but also *IL1β* production, as has been demonstrated in a sepsis cell model (Sheng et al., 2016).

The polymorphism rs1143633 at *IL1β* is an intron variant consisting on a C to T transition. Among the most powerful associations involving this variant, an increased risk with eczema cases has been reported in the context of a genome-wide association study (GWAS) to elucidate shared genetic variants of allergic diseases (Ferreira et al., 2017). As an intron variant close downstream to the nearest splice site (64 bases), the presence of this polymorphism could have an impact on the splicing process and, therefore, on the correct protein synthesis that could alter the levels of *IL1β*. This alteration could potentially affect the efficacy of Tocilizumab since *IL1β* levels have been shown to predict the response to Tocilizumab in rheumatoid arthritis patients (Okano et al., 2016).

*Interleukin one receptor antagonist* (*IL1Ra*) is an anti-inflammatory cytokine that inhibits the pro-inflammatory activity of *IL1α* and *IL1β* by binding, in a competitive manner, to the *IL1* receptor. *IL1RN* gene regulates *IL1Ra* production, and polymorphic variants within this gene have been shown to modify the inflammatory response by modulating *IL1Ra* production (Khazim et al., 2018). In fact, some of these variants have been associated with different inflammatory disorders such as inflammatory bowel disease, SLE, or insulin resistance (Tjernström et al., 1999; Dinarello et al., 2012; Ramírez-Pérez et al., 2017). The SNP rs2234679 is a G to C transversion located at 5′ UTR of the *IL1RN* gene. This polymorphism is part of a haplotype that has been significantly associated with SLE and, consequently, different expression patterns of *IL1RN* isoforms were detected between healthy controls and SLE cases (Tsai et al., 2006).

Genetic variations at UTRs may alter regulatory mechanisms, through the interaction of these UTRs with proteins or even microRNAs, known to modify molecular pathways and cellular processes. The functional consequences of these genetic variations include access to regulators or modulation of mRNA transcription. Taken together, these alterations can potentially lead to disease processes (Steri et al., 2018). Specifically, the results of the current study show that the presence of the CC genotype at *IL1RN* polymorphism associates with an increased risk of mortality in severe COVID-19 patients that are not treated with Tocilizumab. Some studies showed that *IL1Ra* levels are significantly elevated in severe COVID-19 patients (Zhao et al., 2020; Attur et al., 2023), however, other studies did not find such differences (Wilson et al., 2020). To what extent the presence of this genotype influences *IL1Ra* levels or function and, therefore, regulates *IL1β* driven expression of pro-inflammatory cytokines and, ultimately, affects the efficacy of Tocilizumab, deserves further investigation. What is evident from this study is that *IL1* pathway seems to play a pivotal role on the risk of mortality in severe COVID-19 patients, as previously postulated (Attur et al., 2023), and that genetic regulation of these genes could have an impact on the efficacy of Tocilizumab in terms of mortality.

The study presented herein has two main limitations. On the one hand, due to the complexity of finding larger cohorts with the same characteristics as the one in this study, we have not been able to replicate these results in an independent cohort, but rather performed an internal bootstrap validation strategy to give robustness to the findings. Despite this strategy does not provide any information about external validity, it provides a robust estimate of model performance, when performed by taking the appropriate steps during model development, allowing using more data for training and helping avoid overfitting and assess model reproducibility (Steyerberg and Harrell, 2016; Sperrin et al., 2022). On the other hand, our results did not pass the Bonferroni multiple correction test (*p*-value = 0.05/8933 = 5.6e-06), probably due to the limited sample size of the study; however, in addition to the above mentioned bootstrap approach for the internal validation of the models, we also assessed the quality of the study and the comprehensiveness of the results (effect size) before interpreting statistical significance. Therefore, the findings presented in this study should be considered as exploratory.

Although more significant signals were detected, we selected these three variants due to different reasons. On the one hand, rs2834167 (*IL10Rβ*) was selected because was the only missense variant with a moderate impact that was identified and it was previously associated with other immune related diseases. On the other hand, rs2234679 (*IL1RN*) was selected because it belongs to the most important linkage block, together with another 11 SNPs, that we found after the in-depth sequencing, which could be indicative of a robust association; although additional fine mapping and functional studies would be necessary to confirm this last point. Finally, rs1143633 at *IL1β* gene was also selected because of its previously demonstrated implication in COVID-19 disease and due to its belonging to the same gene family as *IL1RN* (rs2234679).

In summary, despite the limitations described above, this study identified a series of genotypes at *IL10Rβ*, *IL1β* and *IL1RN* genes that showed a significant association with the efficacy of Tocilizumab. The implication of these genes in the development of severe COVID-19 disease has been widely demonstrated. If robustly validated in larger external cohorts, the application of these predictive models could help clinicians to identify those severe COVID-19 patients to be treated with Tocilizumab and thus reduce their risk of mortality.

## Conclusions and future perspectives

In this work, we developed a series of predictive models consisted of both clinical and genetic variables able to predict the risk of mortality in severe COVID-19 patients treated with Tocilizumab. The three genotypes have been detected at *IL10Rβ*, *IL1β* and *IL1RN* genes, which have a strong implication in the pathogenesis of severe COVID-19 disease. If robustly validated in larger external cohorts, the genotyping of rs2834167, rs1143633 and rs2234679 polymorphisms could be an adequate therapeutic strategy to predict the efficacy of this drug. In the present study, these findings were validated performing a robust internal validation approach based on a bootstrap strategy.

Fortunately, at this time, the fatal consequences that took place during the onset of COVID-19 pandemic outbreak are far from occur. Those terrible consequences led to the search of appropriate drugs to try to decrease the number of deceased patients through a strategy based on drug repurposing. Among those drugs, Tocilizumab has been approved for use by the WHO. The findings shown in this study and others, should be interpreted as an endorsement of personalized medicine, which is beginning to give such good results in the field of medicine. A large amount of resources must be devoted towards the development of effective precision medicine, and pharmacogenetic studies are key in this process.

## Data Availability

The data presented in the study are deposited in Zenodo repository, available at zenodo.org/records/13692512.

## Glossary

ARDS: Acute respiratory distress syndrome
AUC: Area under the curve
CI: Confidence interval
COVID-19: Coronavirus disease 2019
CRP: C-reactive protein
DNA: Deoxyribonucleic acid
GWAS: Genome-wide association study
ICU: Intensive care unit
IFNAR1: Interferon-α receptor 1
IFNAR2: Interferon-α receptor 2
IFNGR2: Interferon-γ receptor 2
IL10: Interleukin 10
IL10Rβ: Interleukin 10 receptor β
IL1Ra: Interleukin 1 receptor antagonist
IL1RN: Interleukin 1 receptor antagonist
IL1α: Interleukin 1 03B1
IL1β: Interleukin 1 β
IL6: Interleukin 6
LDH: Lactate dehydrogenase
MAF: Minor allele frequency
MERS: Middle east respiratory syndrome
microRNA: Micro ribonucleic acid
mRNA: Messenger ribonucleic acid
NLRP3: Nod-like receptor protein 3
NPV: Negative predictive value
OR: Odds ratio
PAF: Population attributable fraction
PCR: Polymerase chain reaction
PHRED: Phil’s read editor
PPV: Positive predictive value
ROC: Receiver operating characteristic
SARS: Severe acute respiratory syndrome
SARS-CoV-2: Severe acute respiratory syndrome coronavirus 2
SBE: Single base extension
SLE: Systemic lupus erythematosus
SNP: Single nucleotide polymorphism
TNFα: Tumor necrosis factor α
TRIPOD: Transparent reporting of a multivariate rediction model for individual prognosis or diagnosis
UTR: Untranslated region
VCF: Variant call format
WHO: World health organization
